# Probabilistic Dynamic Deployment of Wireless Sensor Networks by Artificial Bee Colony Algorithm

**DOI:** 10.3390/s110606056

**Published:** 2011-06-03

**Authors:** Celal Ozturk, Dervis Karaboga, Beyza Gorkemli

**Affiliations:** Computer Engineering Department, Engineering Faculty, Erciyes University, 38039 Kayseri, Turkey; E-Mails: karaboga@erciyes.edu.tr (D.K.); bgorkemli@erciyes.edu.tr (B.G.)

**Keywords:** artificial bee colony algorithm, wireless sensor networks, dynamic deployment, probabilistic detection model

## Abstract

As the usage and development of wireless sensor networks are increasing, the problems related to these networks are being realized. Dynamic deployment is one of the main topics that directly affect the performance of the wireless sensor networks. In this paper, the artificial bee colony algorithm is applied to the dynamic deployment of stationary and mobile sensor networks to achieve better performance by trying to increase the coverage area of the network. A probabilistic detection model is considered to obtain more realistic results while computing the effectively covered area. Performance of the algorithm is compared with that of the particle swarm optimization algorithm, which is also a swarm based optimization technique and formerly used in wireless sensor network deployment. Results show artificial bee colony algorithm can be preferable in the dynamic deployment of wireless sensor networks.

## 1. Introduction

Wireless sensor networks are used for target tracking, environment monitoring, surveillance and for getting humidity, temperature, light, pressure data, *etc*. and obtaining information about things like the weight, velocity, movement direction of an object in an area of interest [1]. Regardless of hpw these networks are used in these applications, the success of the network is highly dependent on the sensors’ positions, referred to as the deployment of the network. Deciding the positions of the sensors is the main subject of sensor network deployment, and in turn it depends on the desired coverage of the area of interest. In dynamic deployment problem, initially sensors are located in the area with random positions and the sensors change their positions by using the knowledge of others positions, if they are mobile. By these movements, it is tired to increase the coverage rate of the sensors. On the other hand, if the sensors are stationary, they do not have ability to change their positions.

In initial deployment, because of the randomness, generally an effective coverage cannot be obtained. To tackle this problem, various dynamic deployment algorithms have been studied by researchers [2–5]. To improve the coverage of the network, one of the approaches used in these researches is the virtual force (VF) algorithm [6], which works well for WSNs which consist only of mobile sensors [6–8]. In [9], a blackboard mechanism based on ant colony theory was proposed for dynamic deployment of mobile sensor networks. Kukunuru *et al*. used an approach based on particle swarm optimization (PSO) to solve the mobile sensor network coverage problem [10] in which the main objective is to minimize the distance between the neighboring nodes, thus maximizing coverage in the network. These approaches do not consider the stationary sensors which are not able to change their initial positions. However, to save energy and to reduce cost, stationary sensors are widely used in real life network applications. Wang *et al*. considered both stationary and mobile sensors together in WSNs and proposed a new approach based on parallel particle swarm optimization (PPSO) in [11], then they proposed VFCPSO algorithm based on VF algorithm and co-evolutionary particle swarm optimization (CPSO) in [12]. Li and Lei proposed a method of improved particle swarm optimization to solve the deployment problem of WSNs consist of stationary and mobile sensor nodes [13]. Soleimanzadeh *et al*. considered mobile and stationary sensors together as a hybrid network and proposed three dynamic PSO-based deployment algorithms in [14]: PSO-LA, Improved PSO-LA, and Improved PSO-LA with logical movement. In PSO-LA algorithm, PSO and learning automata are hybridized where speed of particles is corrected by using the existing knowledge and the feedback from the actual implementation of the algorithm. To improve the performance of the PSO-LA, Improved PSO-LA algorithm is introduced, regulating movement of a node without an impact from the movement of other mobile nodes and based on the result gained from its previous movement. In the third one, Improved PSO-LA with logical movement, sensors virtually move new positions by calculating their target locations with the same procedure of the Improved PSO-LA, but the real movement of the nodes only happens at the last round after final destinations are determined.

In this study, a new approach for dynamic deployment problem for WSNs is proposed. We considered WSNs which consist of mobile and stationary sensors together. This approach is based on Artificial Bee Colony (ABC) algorithm which is developed by modeling foraging behavior of honey bee swarms [15,16]. It is known that the ABC algorithm works well for numerical optimization problems [17–19]. The ABC algorithm was first tested on dynamic deployment a using binary detection model of wireless sensor networks consisting of all mobile nodes in [20]. Considering the good performance of the algorithm, use of the ABC algorithm will be a proper approach for the sensors in the network to obtain a good coverage in two dimensional space with stationary and mobile nodes. The performance of proposed approach is evaluated in comparison with another swarm based technique, Particle Swarm Optimization (PSO).

We have organized rest of the paper as follows: Section 2 explains dynamic deployment problem of WSNs and sensor detection models, the proposed approach is presented in Section 3 and followed by the simulation results and comparison of PSO algorithm and proposed approach for this problem in Section 4. Finally, Section 5 concludes the paper and discusses the future path of our work.

## 2. WSN Dynamic Deployment Problem and Sensor Detection Model

The performance of a sensor network depends on the positions of the sensors in the area of interest area. Therefore, by responding to all system objectives, deployment of the sensors in the mission space is a problem which is called the coverage control or active sensing problem [21–24]. In the applications which consider coverage, sensors should be deployed to maximize the information that they collect from the area of the interest. In the static version of the problem, after the sensors’ first positioning, there will be no mobility anymore in the network. Optimal locations can be found by using an off-line scheme as a facility location optimization problem. On the other hand, in the dynamic version of the networks, sensors are able to move coordinately in the mission space [25].

In WSNs, sensors can collect information about the area within their detection ranges. They share their information with their neighbor sensors as well with base stations. Therefore, to have an effective detection in a network including communicated sensors with each-other, the covered area should be expanded. In order to increase the ratio of covered area, mobile sensors’ positions changeability property can be used.

Since there is no *a priori* information about the sensing area, initial positions of the sensors are chosen randomly and deployment of sensors on the area of the interest will be obtained dynamically. The sensor field is a two-dimensional grid. Each sensor knows its position. Sensors communicate with others and the mobile ones can change their positions by using the others’ information. Coverage ratio of the WSN is calculated by Equation (1):

(1) $$CR=\frac{\cup c_{i}}{A},i\in S$$

where *c_i_* is the coverage of a sensor *i*, *S* is the set of the nodes, and *A* is the total size of the area of the interest.

There are two sensor detection models in WSNs to find out the effective coverage. One of them is binary detection model which assumes that there is no uncertainty and the other one is probabilistic detection model which gives more realistic results because of using probabilistic terms for deciding the effective coverage of the area [6].

Assuming that, there are *k* sensors in the random deployment stage, each sensor has the same detection range *r*, sensor *s_i_* is positioned at point (*x_i_*,*y_i_*). For any point *P* at (*x*,*y*), Euclidean distance between *s_i_* and *P* is *d*(*s_i_*,*P*). The binary sensor model [26,27] is shown by Equation (2):

(2) $$c_{xy}(s_{i})=\left\{\begin{array}{cc} 1, & \mathrm{if}\;d(s_{i},P)\;<\;r\\ 0, & \mathrm{otherwise} \end{array}\right\}$$

where *c_xy_* (*s_i_*) is the coverage of a grid point *P* by sensor *s_i_*, *d*(*s_i_*,*P*) is Euclidean distance.

While the binary model interests with only detection range, the probabilistic model also considers detection uncertainty range and measuring parameters, which is given by Equation (3) [8]:

(3) $$c_{xy}(s_{i})=\{\begin{array}{cc} 0, & \mathrm{if}\;r+r_{e}\;\leq d(s_{i},P)\\ e^{(-\lambda_{1}\alpha_{1}^{\beta_{1}}/\alpha_{2}^{\beta_{2}}+\lambda_{2}),} & \mathrm{if}\;r-r_{e}\;<\;d(s_{i},P)\;<\;r+r_{e}\\ 1, & \mathrm{if}\;d(s_{i},P)\leq r-r_{e} \end{array}$$

where *β*_1_, *β*_2_, *λ*_1_ are measuring parameters for the detection probability; *α*_1_= *r_e_* – *r* + *d*(*s_i_*,*P*) and *α*_2_ = *r_e_* + *r* − *d*(*s_i_*,*P*); *λ*_2_ is the disturbing effect; *r_e_* (*r_e_* < *r*) is the detection uncertainty range.

In our work, we used the probabilistic sensor detection model. Using this model all of the points in the area are covered with different probabilities. If a point is covered by only one sensor it will have low coverage, so overlapping of the detection areas is very important for compensating for the potential low detection probability of the points which are far from a sensor node. The coverage of the overlapped area *S_ov_* which is overlapped by a set of *k_ov_* sensors is shown in Equation (4) [6]:

(4) $$c_{xy}(S_{ov})=1-\prod_{s_{i}\in S_{ov}}\left(1-c_{xy}(s_{i})\right)$$

To decide the effectiveness of the coverage area, the desired coverage threshold *c_th_* is used as in Equation (5):

(5) $$c_{xy}(S_{ov})\;\;\geq \;\;c_{th}$$

## 3. Dynamic Deployment of Wireless Sensor Networks with Artificial Bee Colony Algorithm

The Artificial Bee Colony (ABC) algorithm, a swarm based intelligent method inspired by modeling the foraging behavior of honey bees, is used for the dynamic deployment problem of WSNs. The aim of the use of optimization technique is to maximize the coverage rate of the network, given with Equation (1) where it is assumed within the network scenario:

- Detection radius of the sensors are all same (*r*),
- All of the sensors have ability to communicate with other sensors,
- WSN consists of both mobile and stationary sensors.

In the ABC algorithm, the position of a food source represents a possible solution to the optimization problem and the nectar amount of a food source corresponds to the quality (fitness) of the associated solution. Therefore, the deployment of the sensors in the sensed area refers a food source (a solution) in the algorithm. The coverage rate of the network, *i.e.*, total covered area, corresponds to the fitness value (nectar) of the solution. In ABC model, artificial bee colonies where the goal of the bees is to find the best solution [28] are formed of three groups of bees: worker bees, onlookers and scouts. A bee waiting on the dance area to determine to choose a food source is an onlooker and a bee goes to the food source visited by it previously is a worker bee. A bee that carries out random search is called a scout.

The steps of the algorithm are:

- Initialize the parameters: detection radius *r*, size of the area of the interest *A*, number of the mobile sensors *m*, number of the stationary sensors *s*, colony size *cs*, maximum number of iterations *MaxNumber*, limit for scout *l*.
- Deploy the *s* stationary sensors randomly.
- Determine the positions of m mobile sensors randomly for each food source *x_i_* of worker bees using Equation (6) where j = 1,2,...,2 m:
  (6) $$x_{ij}=\min_{j}+\mathrm{rand}(0,1)(\max_{j}-min_{j})$$
- Evaluate the population
- *c* = 0
- **REPEAT**
  - Produce new solutions *υ_i_* in the neighborhood of *x_i_* for the worker bees using Equation (7):
    (7) $$v_{ij}=x_{ij}+\phi_{ij}(x_{ij}-x_{kj})$$
    *k* is a solution in the neighborhood of *i (k ≠ i)*, *ϕ* is a random number in the range [−1,1] and *j* is the randomly selected mobile sensor’s position.
  - Check *υ_ij_* for staying in the bounds of the area.
  - Apply the greedy selection process between *x_i_* and *υ_i_*.
  - Calculate the probability values *P_i_* for the solutions *x_i_* by means of their fitness values using Equation (8).
    (8) $$P_{i}=\frac{0.9\times fit_{i}}{fit_{best}}+0.1$$
  - Produce the new solutions *υ_i_* for the onlooker bees from the solutions *x_i_*, selected depending on *P_i_* and evaluate them.
  - Apply the greedy selection process for the onlookers between *x_i_* and *υ_i_*.
  - Memorize the best solution achieved yet.
  - Determine the abandoned solution, if exists, replace it with a new randomly produced solution for the scout, using Equation (6).
  - *c* = *c* + 1
- **UNTIL** *c* = *MaxNumber*.

**Figure 1.**
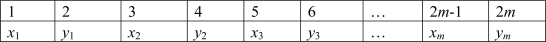
Solution array.

Each solution represents an array that has 2 m items. Figure 1 shows a solution array. Items of the solution array are (x, y) positions of the mobile sensors in the network.

## 4. Simulation Results

In this work, the performance of the ABC algorithm on dynamic deployment of WSNs is compared with the results of the Particle Swarm Optimization (PSO) algorithm. In the PSO algorithm, velocity and position of the particles are updated by Equation (9) and Equation (10) as in [13]:

(9) $$v_{ij}(c+1)=\omega (c)\times v_{ij}(c)+c_{1}r_{1i}(c)\left(y_{ij}(c)-x_{ij}(c)\right)+c_{2}r_{2i}(c)\left(\hat{\text{y}}(c)-x_{ij}(c)\right)$$

(10) $$x_{ij}(c+1)=x_{ij}(c)+v_{ij}(c+1)$$

where *c*_1_ and *c*_2_ are acceleration constants, *r*_1_*_i_*(*c*) and *r*_2_*_i_*(*c*) are random numbers in range [0,1]. *x_ij_*(c) and *v_ij_*(c) represents the position and velocity of *i_th_* particle in *j_th_* dimension at time *c*, *y_i_*(*c*) is the local best position of *i_th_* particle and *ŷ*(*c*) is the global best position. The inertia weight ω *(c)* at time *c* is set by using Equation (11):

(11) $$\omega (c)=0.9-\frac{c}{MaxNumber}\times 0.5$$

where *MaxNumber* is the maximum number of cycles.

In the simulations, a wireless sensor network including 20 mobile and 80 stationary sensors is simulated as in [13]. Detection radius of the each sensor *r* is 7 m, the range detection error *r_e_* is 0.5 *r* = 3.5 m, size of the area which is a square region *A* is 10,000 m^2^, the probabilistic detection parameters *λ*_1_ = 1, *λ*_2_ = 0, *β*_1_ = 1, *β*_2_ = 0.5.

The ABC algorithms’ control parameters are set as follows: the colony size *cs* is 20, limit parameter *l* for the scout is taken 100. PSO algorithms’ swarm size is 20 and the acceleration constants are set *c*_1_ = *c*_2_ = 1 as in [13].

To observe the performance of the algorithms, the scenario is run 30 times, each of 1,000 iterations with random initialization. However, to make a reliable comparison the first solution sets of the ABC and PSO algorithms are taken. The average coverage rates of the algorithms are given in Table 1 by the mean values. In the Table, standard deviation of 30 runs, the best and the worst of the runs are reported.

**Figure 2.**
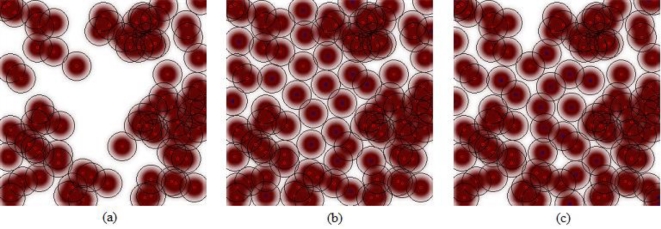
**(a)** Initial deployment of stationary sensors. **(b)** Final deployment of ABC algorithm (703th iteration). **(c)** Final deployment of PSO algorithm (901th iteration).

As seen from Table 1, the ABC algorithm is more successful than the PSO algorithm for the dynamic deployment problem of WSNs using a probabilistic detection model. In addition, the simulation results show that the deployments found by ABC are better than the deployments found by PSO for all of the 30 independent runs which are started with the same initial deployment. Figure 2 shows one of the initial deployments of the stationary sensors, the final deployment of proposed ABC approach and final deployment of PSO algorithm for an independent run. It should be noticed that for a given simulation result, ABC found the best deployment in the 703th iteration, on the other hand the final deployment of PSO algorithm is achieved in the 901th iteration.

**Figure 3.**
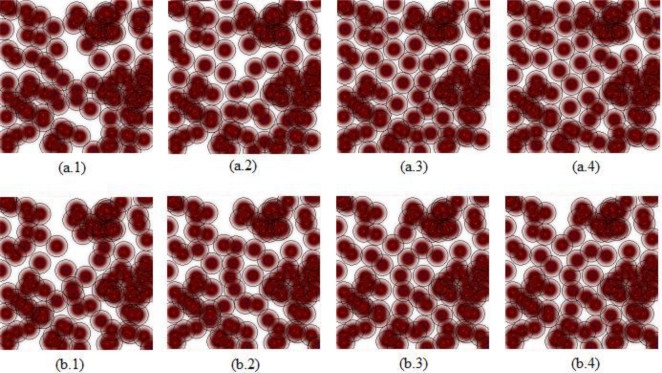
Best solutions of ABC: **(a.1)** iteration #50, **(a.2)** iteration # 100, **(a.3)** iteration # 500, **(a.4)** iteration # 1000. Best solutions of PSO: **(b.1)** iteration #50, **(b.2)** iteration # 100, **(b.3)** iteration # 500, **(b.4)** iteration # 1000.

**Figure 4.**
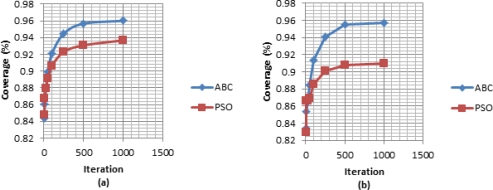
Development of the populations through the iterations for ABC and PSO algorithms: **(a)** the average of 30 runs, **(b)** the most difference in a run.

To observe the development of the best solutions for the algorithms through the iterations Figures 3 and 4 are shown. In Figure 3, the convergences of two algorithms are shown by coverage rate for the iterations: iteration number 50, iteration number 100, iteration number 500, and iteration number 1000. Figure 4 including development graphics of the average and best of the populations through the iterations for ABC and PSO algorithms, demonstrates that ABC algorithm finds better deployments than PSO algorithm in fast manner. Figure 4(a) demonstrates the convergence graphic of mean of best solutions of 30 runs and Figure 4(b) demonstrates the convergence graphic of a run in which the difference of ABC and PSO algorithms is the most. It should be mentioned that in all runs the algorithms are started with the same initial deployment to make a fair comparison. The execution times of the algorithms are noted on a PC with 2.8 GHz Core 2 Duo processor and 6.0 GB RAM as: 98.46 min/run for the PSO algorithm and 98.83 min/run for the ABC algorithm.

## 5. Conclusions

In this paper, the ABC algorithm is applied to the dynamic deployment problem in WSNs within the scenario of mobile and stationary sensors, which is based on a probabilistic detection model. The performance of the algorithm is compared with the PSO algorithm, which is a well-known swarm based optimization technique. In the simulations, a similar network scenario which is studied in the literature is tried to be used to make comparison. Simulation results show that the ABC algorithm obtains better deployments for WSNs than the PSO algorithm. As a future work, we are planning to study the usage and performance of the ABC algorithm not only in the dynamic deployment of WSNs, but also for other optimization issues like localization and routing.

## Figures and Tables

**Table 1.** Probabilistic Dynamic Deployment Results.

	**Initial coverage of stationary sensors**	**PSO**	**ABC**

**Mean**	0.7436	0.9368	0.9601
**Std**	0.0224	0.0128	0.0078
**Best**	0.7888	0.9581	0.9752
**Worst**	0.6975	0.9094	0.9365
